# Endotoxin promotes neutrophil hierarchical chemotaxis via the p38-membrane receptor pathway

**DOI:** 10.18632/oncotarget.12093

**Published:** 2016-09-17

**Authors:** Xu Wang, Weiting Qin, Yisen Zhang, Huafeng Zhang, Bingwei Sun

**Affiliations:** ^1^ Department of Burn and Plastic Surgery, Affiliated Hospital, Jiangsu University, Zhenjiang, Jiangsu Province, China

**Keywords:** neutrophil, endotoxin, chemotaxis, membrane receptor, p38

## Abstract

Neutrophils are the most abundant leukocytes in peripheral blood and play critical a role in bacterial infection, tumor immunity and wound repair. Clarifying the process of neutrophil chemotaxis to target sites of immune activity has been a focus of increased interest within the past decade. In bacterial infectious foci, neutrophils migrate toward the bacterial-derived chemoattractant N-formyl-Met-Leu-Phe (fMLP) and ignore other intermediary chemoattractants to arrive at the area of infection. Using an under agarose chemotaxis assay, we observed that the bacterial fMLP-induced neutrophil chemotaxis signal overrode interleukin 8 (IL-8)- and leukotriene B4 (LTB4)-induced chemotaxis signals. Moreover, in the presence of bacterial lipopolysaccharide (LPS), the fMLP-induced hierarchical chemotaxis signal was enhanced. Further studies revealed that LPS increased the membrane expression of the fMLP receptor, formyl peptide receptor 1 (FPR1). However, expression levels of the membrane receptors for IL-8 and LTB4 were decreased by LPS administration. A human Phospho-mitogen-activated protein kinase (MAPK) proteome array showed that the p38 pathway was significantly activated by LPS stimulation. Moreover, p38 was responsible for the altered expression of neutrophil membrane chemoattractant receptors. Inhibition of neutrophil p38 restored LPS-improved hierarchical chemotaxis. Taken together, these data indicate that endotoxin promotes neutrophil hierarchical chemotaxis via the p38-membrane receptor pathway.

## INTRODUCTION

Neutrophils are powerful effector cells for clearing bacterial infection and mediating tumor immunity and wound repair [1, 2]. Neutrophils function as the first line of defense against bacterial infection [3]. Once they recognize the signals of infection, neutrophils rapidly transmigrate to the infected tissue through vascular endothelial cells [4]. To reach and eliminate bacterial pathogens, neutrophils must undergo chemotaxis toward the end-target bacterial chemoattractant N-formyl-Met-Leu-Phe (fMLP) and ignore other stromal cells or macrophage-generated cytokines and leukotrienes [5]. This course is called “hierarchical neutrophil chemotaxis” [6]. However, considering the complexities of inflammatory interstitial tissue environments, other inflammatory stimuli released from immune cells or bacterial debris may also play important roles during neutrophil chemotaxis.

LPS exists in the outer membrane of most Gram-negative bacteria [7]. In the infectious foci, LPS is released from dead Gram-negative bacteria to local microenvironments and transported to tissues and organs. LPS itself is not intrinsically harmful. Instead, by activating toll-like receptor 4 (TLR4) in immune cells, LPS displays a potent ability to induce inflammatory responses [8] and stimulate neutrophils. Administration of LPS promotes neutrophil degranulation, adhesion and reactive oxygen species (ROS) generation [9, 10]. Neutrophil apoptosis can also be delayed by LPS treatment [11]. However, the effect of LPS on hierarchical neutrophil chemotaxis remains unclear.

The present study used under agarose chemotaxis assays to mimic the dense interstitial environments for neutrophil migration. Bacterial LPS was found to promote hierarchical neutrophil chemotaxis. Flow cytometry (FCM) analyses revealed that chemoattractant receptors may be the potential targets of LPS. p38 pathway activation was demonstrated by human Phospho-mitogen-activated protein kinase (MAPK) array and was responsible for the regulation of chemoattractant receptors. The inhibition of p38 depleted the effects of LPS treatment on neutrophil chemotaxis.

## RESULTS

### FMLP-induced chemotaxis signals override other chemoattractants

**Figure 1:**
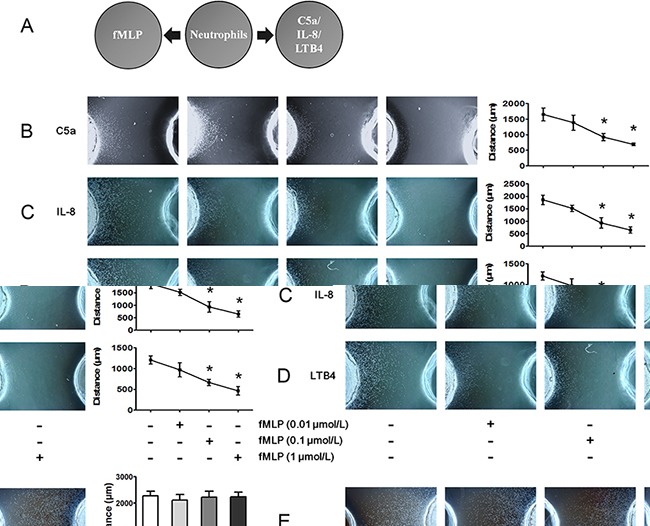
FMLP-induced chemotaxis signal overrides that of other chemoattractants **A.** The under agarose chemotaxis assay was set up as shown. The neutrophil suspension was placed in the middle well, and FMLP was placed in the left well. C5a, IL-8 or LTB4 was placed in the right well. **B-D.** Treatments of 0.01, 0.1 or 1 μmol/L fMLP were placed in the left well, and 1 μmol/L C5a, 1 μmol/L IL-8 or 0.1 μmol/L LTB4 were added to the right wells. Representative neutrophil chemotaxis photos are shown, and chemotaxis distances were calculated. The results show that fMLP 0.1 and 1 μmol/L significantly inhibited C5a-, IL-8- and LTB4-induced neutrophil chemotaxis. **E.** High concentrations of C5a (10 μmol/L), IL-8 (10 μmol/L) and LTB4 (1 μmol/L) exerted no effect on 0.1 μmol/L fMLP-induced neutrophil chemotaxis. The data are expressed as the mean ± SD, n=5 for each group. ^*^P < 0.05 compared with the control group.

The under agarose chemotaxis assay was used to investigate hierarchical neutrophil chemotaxis. The schematic illustration of the under agarose chemotaxis assay is shown in Figure 1A. FMLP is a potent chemotactic peptide for human neutrophils and is recognized as bacterially derived end target chemoattractant. In the present study, we found that 0.1 and 1 μmol/L fMLP inhibited C5a-, IL-8- and LTB4-induced neutrophil chemotaxis (Figure 1B–1D). However, neutrophil chemotaxis to fMLP was not affected by C5a, IL8 or LTB4 even when the concentrations of chemoattractants were 10-fold higher than normal (Figure 1E).

### Low concentrations of LPS inhibit C5a-, IL-8- and LTB4-induced neutrophil chemotaxis but not fMLP-induced neutrophil chemotaxis

**Figure 2:**
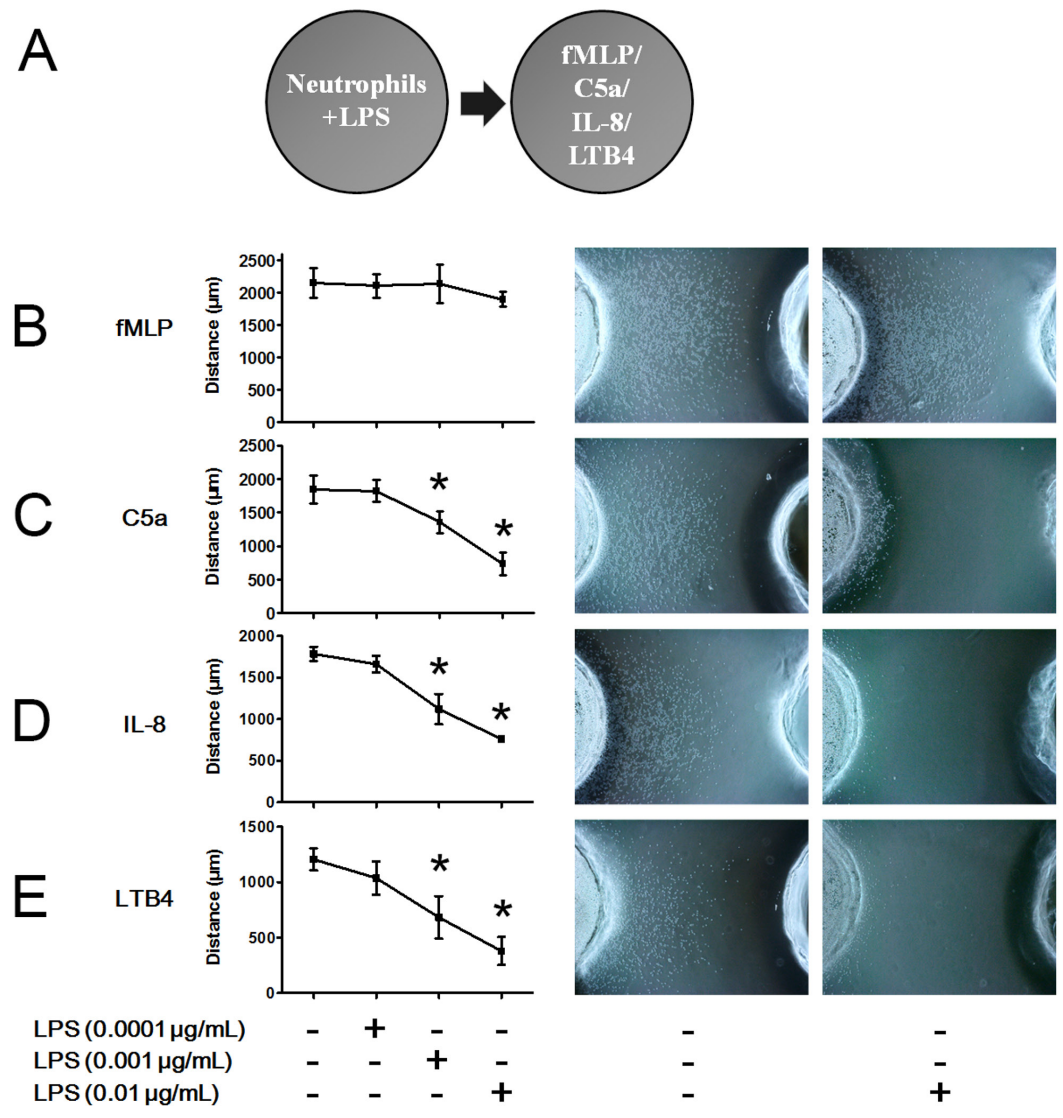
FMLP-induced chemotaxis is less sensitive to LPS treatment than other chemoattractants **A.** The under agarose chemotaxis assay was set as shown. The neutrophil suspension in the presence or absence of LPS was placed in the left well. Treatments of 0.01 μmol/L fMLP, 1 μmol/L C5a, 1 μmol/L IL-8 or 0.1 μmol/L LTB4 were placed in the right well. **B.** LPS did not significantly inhibit fMLP-induced neutrophil chemotaxis. **C-E.** Neutrophil chemotaxis distances of C5a, IL-8 and LTB4 were markedly inhibited by 0.001 and 0.01 μg/mL LPS stimulation. Representative neutrophil chemotaxis photos are shown. The data were expressed as the mean ± SD, n=5 for each group. ^*^P < 0.05 compared with the control group.

LPS is an important inflammatory mediator found on the outer membranes of Gram-negative bacteria. In infectious foci, LPS is shed to active TLR4 in immune cell membranes and elicit inflammatory responses. The schematic illustration of the under agarose chemotaxis assay is shown in Figure 2A. To explore the effects of LPS on neutrophil chemotaxis, we performed the under agarose chemotaxis assays shown in Figure 2A and found inconsistent effects of LPS on fMLP-, C5a-, IL-8- and LTB4-induced neutrophil chemotaxis. FMLP-induced neutrophil chemotaxis distances were not inhibited after stimulation with 0.0001, 0.001 or 0.01 μg/mL LPS (Figure 2B). When the concentrations of LPS were increased to 0.1-1 μg/mL, fMLP-induced neutrophil chemotaxis was compromised. However, the neutrophil chemotaxis distances following treatment with C5a, IL-8 and LTB4 were markedly inhibited by 0.001 and 0.01 μg/mL LPS stimulation (Figure 2C–2E). These results indicated that fMLP-induced neutrophil chemotaxis is less sensitive to LPS stimulation than are other chemoattractants.

### LPS promotes hierarchical neutrophil chemotaxis

**Figure 3:**
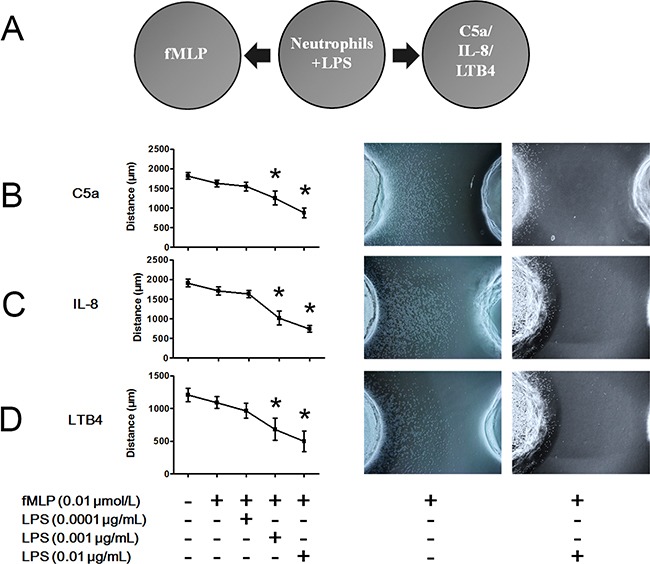
LPS improves fMLP-involved hierarchical neutrophil chemotaxis **A.** The under agarose chemotaxis assay was set as shown. Neutrophil suspension in the presence or absence of LPS was placed in the middle well. Treatment of 0.01 μmol/L fMLP was placed in the left well. Treatments of 1 μmol/L C5a, 1 μmol/L IL-8 or 0.1 μmol/L LTB4 were placed in the right well. **B-D.** Treatment with 0.01 μmol/L fMLP slightly but not significantly inhibited C5a-, IL-8- or LTB4-induced neutrophil chemotaxis. However, in the presence of 0.001 or 0.01 μg/mL LPS, the inhibitory effect of fMLP was enhanced. Representative neutrophil chemotaxis photos are shown. The data are expressed as the mean ± SD, n=5 for each group. ^*^P < 0.05 compared to the control group.

To explore the effects of LPS on hierarchical neutrophil chemotaxis, we performed the under agarose chemotaxis assay, as shown in Figure 3A. As previously mentioned, 0.01 μmol/L fMLP slightly but not significantly decreased C5a-, IL-8- and LTB4-induced neutrophil chemotaxis (Figure 3B–3D). When LPS was administered to neutrophils, the inhibitory effect of fMLP on C5a-, IL-8- and LTB4-induced neutrophil chemotaxis was significantly enhanced in a dose-dependent manner. Thus, hierarchical neutrophil chemotaxis was enhanced by LPS administration.

### LPS is a potent activator of the p38 signaling pathway

**Figure 4:**
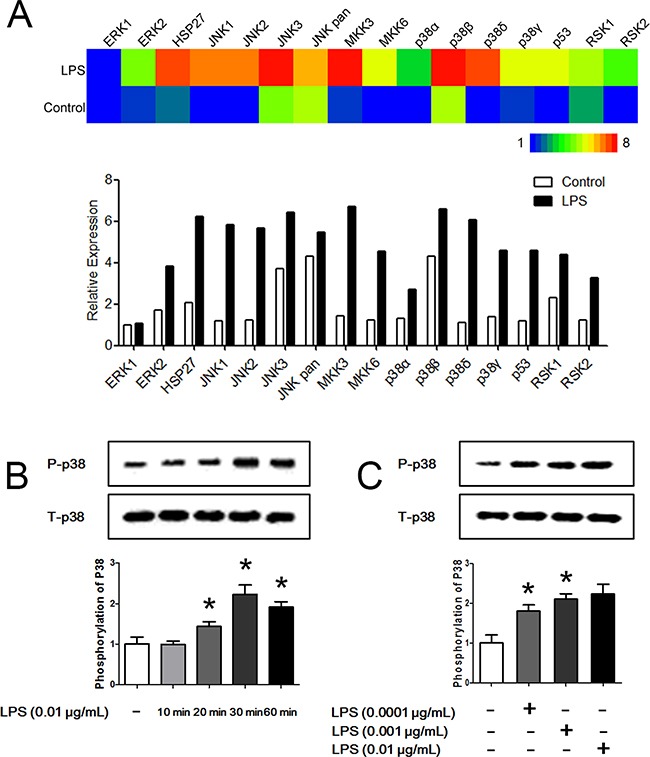
LPS promotes the phosphorylation of p38 **A.** Neutrophils were stimulated with 0.01 μg/mL LPS for 45 min. A Phospho-MAPK proteome array showed that MAPK pathways were significantly activated by LPS. Heat map is showed in the up panel and relative expression values are showed in the down panel. **B.** Neutrophil p38 was significantly phosphorylated after administration of 0.01 μg/mL LPS for 20 min to 60 min. Treatment for 30 min obtained the most significant phosphorylation effect. **C.** When neutrophils were stimulated with LPS for 30 min, LPS administration markedly phosphorylated p38 in a dose-dependent manner. Treatment with 0.01 μg/mL LPS obtained the most significant phosphorylation effect. The data were expressed as the mean ± SD, n=5 for each group. ^*^P < 0.05 compared to the control group.

MAPK pathways are an evolutionarily conserved family of serine/threonine protein kinases that transduce extracellular signals to the machinery that controls fundamental cellular processes, such as proliferation, differentiation, migration, stress responses and survival. MAPK pathways have been shown to play a key role in neutrophil phagocytosis, apoptosis and chemotaxis. To explore the potential targets of LPS on hierarchical neutrophil chemotaxis, neutrophils were stimulated with LPS for 45 min, and the MAPK pathways were analyzed using a Phospho-MAPK proteome array. We found that neutrophil MAPK pathways are profoundly activated by LPS administration (Figure 4A). Notably, all of the p38 isoforms including p38α, p38β, p38δ and p38γ, were significantly phosphorylated. Moreover, their upstream kinases MKK3 and MKK6 as well as the downstream kinase p53 were activated. Western Blot analysis also showed that p38 was significantly phosphorylated after LPS administration (Figure 4B). A 30-min LPS treatment resulted in the greatest extent of phosphorylation. When neutrophils were stimulated with LPS for 30 min, LPS administration markedly phosphorylated p38 in a dose-dependent manner (Figure 4C).

### Bidirectional regulation of LPS on chemoattractant receptors via p38 MAPK

**Figure 5:**
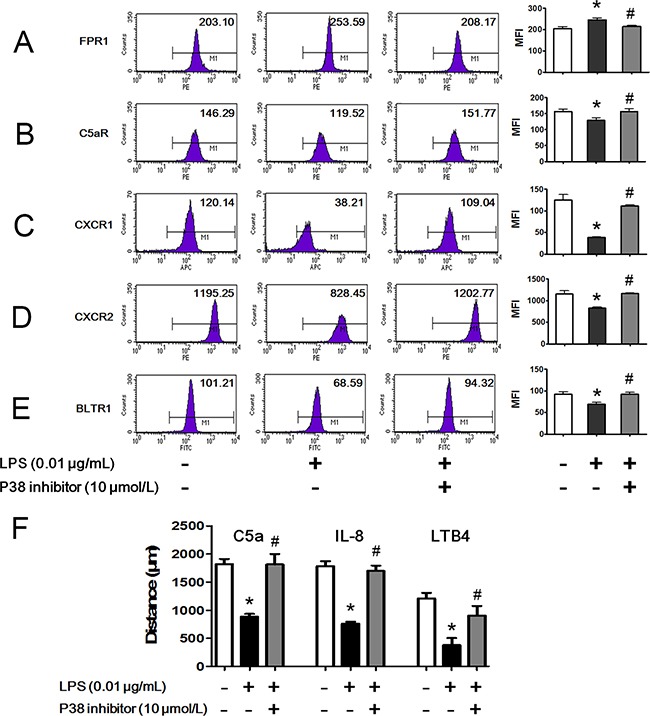
Bidirectional regulation of LPS on chemoattractant receptors via p38 MAPK Neutrophils were stimulated with 0.01 μg/mL LPS for 45 min in the presence or absence of 10 μmol/L p38 inhibitor. **A.** Membrane chemoattractant receptors were detected by FCM. Expression of membrane receptor of fMLP, FPR1, was increased by LPS stimulation. p38 inhibitor restored the increment. **B-E.** Expressions of membrane receptors of C5a (C5aR), IL-8 (CXCR1, CXCR2) and LTB4 (BLTR1) were decreased by LPS stimulation. p38 inhibitor restored the decrements. **F.** LPS inhibited neutrophil chemotaxis toward C5a, IL-8 and LTB4. In the presence of p38 inhibitor, the inhibitory effect of LPS was weakened. The data were expressed as the mean ± SD, n=5 for each group. ^*^P < 0.05 compared to the control group, ^#^*P* < 0.05 compared to the LPS group.

As membrane expression levels of chemoattractant receptors were critical to neutrophil chemotaxis, we detected the membrane expression of different chemoattractant receptors after LPS stimulation with FCM. We found that membrane expression of the fMLP receptor FPR1 was significantly increased after 0.01 μg/mL LPS stimulation for 45 min (Figure 5A). On the contrary, C5a receptor (C5aR), CXC chemokine receptor 1 (CXCR1), CXCR2 and LTB4 receptor 1 (BLTR1), which are receptors for C5a, IL-8 and LTB4, respectively, were internalized after 0.01 μg/mL LPS stimulation for 45 min (Figure 5B–5E). p38 MAPK is a key signal molecule for regulating neutrophil chemotaxis, adhesion and transmigration but whether p38 influences the effects of LPS on chemoattractant receptors remains unknown. The results showed that the bidirectional effects of LPS on chemoattractant receptors were abolished in the presence of the p38 inhibitor SB239063. In addition, the p38 inhibitor restored the LPS inhibition of C5a-, IL-8- and LTB4-mediated neutrophil chemotaxis (Figure 5F).

### Distributions of LPS-stimulated neutrophil chemoattractant receptors

**Figure 6:**
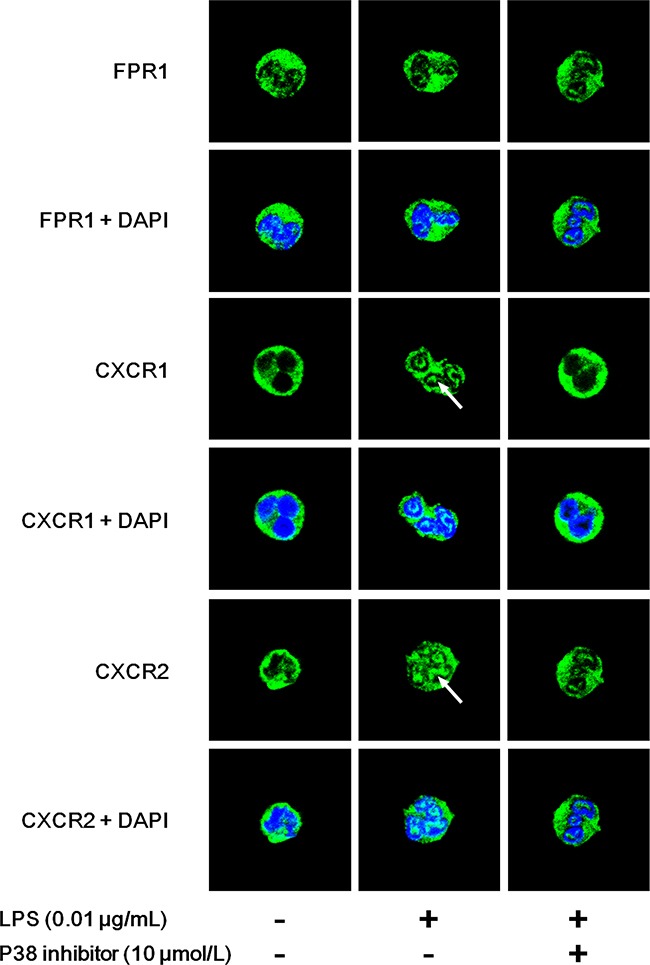
Distributions of LPS-stimulated neutrophil chemoattractant receptors Neutrophils were stimulated with 0.01 μg/mL LPS for 45 min in the presence or absence of 10 μmol/L p38 inhibitor. Distributions of FPR1, CXCR1 and CXCR2 were observed by laser confocal scanning microscopy. n=5 for each group and representative images were shown. FPR1 was distributed mainly in the plasma membranes of untreated neutrophils. LPS administration exerted little effect on FPR1 expression regardless of the presence of p38 inhibitor. However, obvious translocations from the plasma membrane to cytoplasm of CXCR1 and CXCR2 (indicated by arrow) were observed after LPS stimulation. The p38 inhibitor reserved the translocations of CXCR1 and CXCR2.

FCM assays showed the bidirectional effects of LPS on chemoattractant receptors. We further observed the distribution of neutrophil chemoattractant receptors using laser confocal scanning microscopy (LCSM). The unreduced receptor FPR1 and two markedly reduced receptors CXCR1 and CXCR2 were detected. The results of LCSM were consistent with those obtained by FCM. We found that FPR1 was distributed mainly in the plasma membranes of untreated neutrophils (Figure 6). LPS administration exerted little effect on FPR1 expression regardless of the presence of the p38 inhibitor (Figure 6). However, obvious translocations of CXCR1 and CXCR2 from the plasma membrane to the cytoplasm (indicated by arrow) were observed after LPS stimulation. The p38 inhibitor reversed the translocations of CXCR1 and CXCR2.

### p38 participates in LPS enhanced hierarchical neutrophil chemotaxis

**Figure 7:**
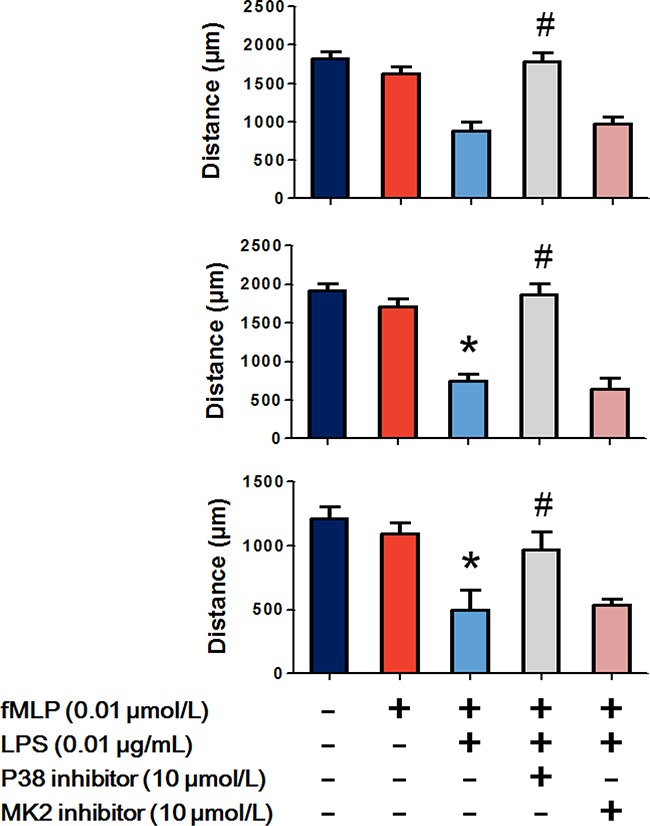
p38 participates in LPS improved hierarchical neutrophil chemotaxis The results of the under agarose chemotaxis assay showed that fMLP 0.01 μmol/L treatment slightly but not significantly decreased C5a-, IL-8- and LTB4-induced neutrophil chemotaxis. LPS 0.01 μg/mL significantly enhanced the inhibitory effect of fMLP, which was then reverted by the p38 inhibitor but not the MK2 inhibitor. The data are expressed as the mean ± SD, n=5 for each group. ^*^P < 0.05 compared to the control group, ^#^*P* < 0.05 compared with the fMLP+LPS group.

As p38 regulated the bidirectional effects of LPS on chemoattractant receptors, we further examined the effect of p38 on LPS enhanced hierarchical neutrophil chemotaxis. Figure 7 shows that 0.01 μmol/L fMLP slightly but not significantly decreased C5a-, IL-8- and LTB4-induced neutrophil chemotaxis. Treatment with 0.01 μg/mL LPS significantly enhanced the inhibitory effect of fMLP, which was then reversed by the p38 inhibitor SB239063. MAPK-activated protein kinase-2 (MK2) downstream of the p38 and the p38/MK2 pathway was found to regulate neutrophil chemotaxis. We observed that the MK2 inhibitor PF-3644022 failed to recover neutrophil chemotaxis.

## DISCUSSION

Neutrophils are the first defensive line of immune cells against bacterial pathogen infection [3, 12, 13]. Neutrophils can eliminate pathogens by phagocytosis, degranulation and neutrophil extracellular traps (NETs) when recruited to the infectious foci [14–16]. To reach the pathogens, neutrophils must first interact with vascular endothelial cells via selectin- and integrin-dependent pathways and transmigrate through vascular endothelial barriers into interstitial tissues. In the tissues separate from pathogens, macrophages and stromal cells release endogenous intermediate chemoattractants to guide neutrophil migration. Following the intermediate chemoattractant gradients, neutrophils approach the infectious foci. Subsequently, neutrophils sense the bacterial generated end-target chemoattractants that are able to override endogenous signals [6, 17, 18]. Hierarchical chemotaxis is critical for neutrophils to reach the bacterial infectious foci by discriminating between the bacterial signals and intermediate signals [6, 17]. PTEN has been proven to be a key regulator involved in this process via the inhibition of phosphatidylinositol-3-OH kinase (PI(3)K) phosphatase [18, 19]. However, given the complicated microenvironments of bacterial infection, we speculate that other regulators may also participate in the hierarchical neutrophil chemotaxis. In the present study, we found that the bacterial-associated chemoattractant fMLP inhibited C5a-, IL-8- and LTB4-induced neutrophil chemotaxis using the under agarose chemotaxis assay. Although C5a was also recognized as an end-target chemoattractant activated by bacteria [20], fMLP was found to be more attractive for neutrophils. On the contrary, C5a, IL-8 and LTB4 failed to affect fMLP-induced neutrophil chemotaxis. Under agarose chemotaxis assay demonstrates that fMLP-induced neutrophil chemotactic signal is able to override other chemoattractants-induced chemotactic signals. It is suggested that at the site of bacterial infection, neutrophils have the ability to distinguish and prioritize chemotactic cues so that selectively ignore potential distractive molecules to reach the site of pathogens.

LPS is a potent inflammatory signal released from dead Gram-negative bacteria [21]. Through the activation of TLR4 in immune cells, LPS provokes strong inflammatory responses [22, 23]. Previous reports have shown that LPS primes neutrophils for enhanced neutrophil oxidative bursts, degranulation and adhesion. However, the effects of LPS on neutrophil chemotaxis are controversial. Jie *et al.* and Shinjiro *et al.* found that LPS augmented chemokine-induced neutrophil migration by modulating cell surface expression of chemokine receptors and elastase [24]. Adil *et al.* proved that LPS was a disrupter of neutrophil chemotaxis [25]. Differences in doses and timing of LPS stimulation, experimental chemotaxis models and chemoattractants may contribute to these contradictions. In addition, the effect of bacterial LPS on hierarchical neutrophil chemotaxis still remains unknown. To explore the potential mechanisms of LPS on hierarchical neutrophil chemotaxis, we first assessed LPS-stimulated neutrophil chemotaxis toward a single chemoattractant. The results revealed that neutrophil chemotaxis to fMLP was not affected by 0.01 μg/mL LPS. However, C5a-, IL-8- and LTB4-induced neutrophil chemotaxis were markedly inhibited. LPS exerted no effect on bacterial chemoattractants but inhibited exogenous chemoattractants. Therefore, we suggest that the inconsistent effects of LPS on different chemoattractants resulted in enhanced hierarchical chemotaxis. It is worth noting that when LPS concentrations were raised to 0.1 and 1 μg/mL, neutrophil chemotaxis toward all chemoattractants was inhibited. The mechanisms of high concentrations of LPS on neutrophil chemotaxis are unclear and not discussed in the present study. Next, we further detected the effects of LPS on hierarchical neutrophil chemotaxis. The results showed that LPS administration enhanced the inhibitory effects of fMLP on neutrophil chemotaxis toward C5a, IL-8 and LTB, which indicated that hierarchical chemotaxis was promoted by LPS. Given that the LPS released from bacteria in tissues close to infectious foci, neutrophils take advantage of LPS signals to promote hierarchical chemotaxis and move toward pathogens more effectively.

Various molecular mechanisms have been reported to mediate neutrophil chemotaxis, and chemoattractant receptor internalization is the most studied because of its crucial role in initiating neutrophil migration. Therefore, we assessed neutrophil membrane receptors for fMLP, C5a, IL-8 and LTB4 after LPS stimulation. The FCM results showed that membrane expression of FPR1 was slightly increased, but membrane expression levels of C5aR, CXCR1, CXCR2 and BLTR1 were significantly decreased. LCSM images also showed the translocations of CXCR1 and CXCR2 to the cytoplasm. These results were consistent with previous chemotaxis analyses, which indicated that altered internalization of membrane chemoattractant receptors might be a potential target of LPS.

MAPKs are a highly conserved family of kinases that are specific to the amino acids serine and threonine. MAPKs are essential for cellular responses to a diverse array of stimuli, such as bacterial toxins, osmotic stress, heat shock and cytokines. The effects of MAPKs on neutrophil chemotaxis have been well recognized; however, whether MAPKs are involved in the regulations of LPS on neutrophil chemoattractant receptors are still unclear. Based on the results of Phospho-MAPK proteome array, we observed that neutrophil MAPKs were profoundly activated by bacterial LPS treatment. Four isoforms of p38 MAPKs as well as their upstream and downstream molecules were significantly phosphorylated following LPS stimulation. Using FCM, we observed that LPS-induced increased expression membrane FPR1 was prevented by a p38 inhibitor. Meanwhile, the LPS-induced decreased expression levels of C5aR, CXCR1, CXCR2 and BLTR1 were also prevented by the p38 inhibitor. The p38 inhibitor restored the inhibitory effects of LPS on C5a-, IL-8- and LTB4-induced neutrophil chemotaxis. Moreover, the improved hierarchical neutrophil chemotaxis was also abolished by the p38 inhibitor. The p38/MK2 pathway has been shown to participate in neutrophil polarization and chemotaxis [26, 27]. However, MK2 might not be involved in this process because an MK2 inhibitor exerted no effect on neutrophil chemotaxis. These data demonstrated that p38 was required for LPS-improved hierarchical neutrophil chemotaxis by bidirectional regulation of chemoattractant receptors. Considering that chemoattractant receptors are a group of seven transmembrane, G protein coupled receptors (GPCRs), the structural characteristics in intracellular domains control the internalization of chemoattractant receptors and may be causes of the p38-induced bidirectional effects [28]. Liu et al. found that phosphorylation of Ser342 in FPR1 by p38 prevented the receptor from internalization [29]. Besides, p38 is involved in the down-regulation of neutrophil CXCR1 and CXCR2 during human endotoxemia but the detailed mechanism remains to be discovered [30]. Further discoveries are still needed to explore the potential targets of p38 on chemoattractant receptors.

**Figure 8:**
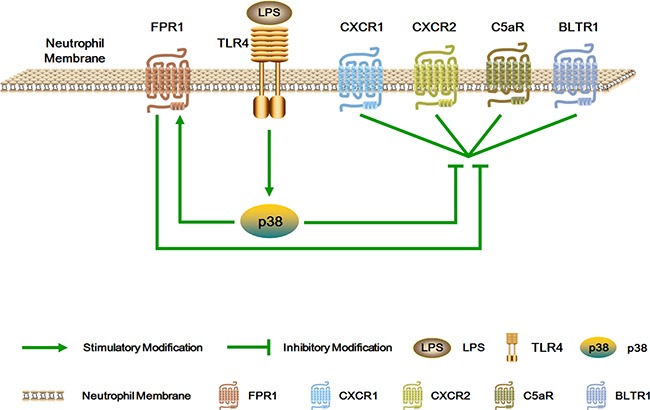
Schematic illustration of the mechanism of the inhibitory effect of fMLP on C5a-, IL-8- and LTB4-induced neutrophil chemotaxis LPS promotes the inhibitory effect of fMLP on C5a-, IL-8- and LTB4-induced neutrophil chemotaxis via p38 activation. The promoted hierarchical neutrophil chemotaxis may facilitate neutrophil location and killing of pathogens at sites of bacterial infection.

In summary, these results indicate that LPS promotes the inhibitory effect of fMLP on C5a-, IL-8- and LTB4-induced neutrophil chemotaxis via p38 activation (Figure 8). The promoted hierarchical neutrophil chemotaxis may facilitate neutrophil location and killing of pathogens at sites of bacterial infection.

## MATERIALS AND METHODS

### Ethics statement

The Medical Ethical Committee of Jiangsu University approved the study. After written informed consent, blood specimens were extracted from healthy drug-free donors' cubital veins. Consent for the use of these samples was given by the Medical Ethical Committee of Jiangsu University. All experiments were performed in accordance with the approved guidelines.

### Materials

LPS, FBS, protease inhibitor cocktail, Ficoll-Paque and RIPA buffer were obtained from Sigma-Aldrich (St. Louis, MO, USA). Agarose, HBSS, RPMI 1640 were obtained from Thermo Scientific (Waltham, MA, USA). P38 inhibitor, MK2 inhibitor, p38 antibodies were purchased from Cell Signaling Technology (Boston, MA, USA). FCM antibodies were obtained from BD Bioscience (San Jose, CA, USA).

### Preparation of human neutrophils

Blood was obtained from the venous blood of healthy volunteers. Neutrophils were isolated as previously described [31]. Briefly, blood was mixed 1:1 with 3% dextran to sediment erythrocytes. The leukocyte fraction was removed and layered onto Ficoll-Paque. The cell solution was spun at 400 g for 30 minutes at room temperature with no brake. Then, neutrophils were collected after RBCs were removed by low osmolality. The neutrophils were suspended in HBSS with Ca^2+^ and Mg^2+^ + 1% FBS at 1.0 × 10^7^ cells/mL. Cell viability (>98%) was monitored by trypan blue exclusion, and the purity (>98%) was verified by Wright staining.

Neutrophils were administered with indicated doses of LPS and chemotaxis was assessed. The p38 inhibitor and MK2 inhibitor were pre-incubated with neutrophils for 30 min before the above-listed interventions.

### Under agarose neutrophil chemotaxis assay

The under agarose neutrophil chemotaxis assay was performed as previously described [32]. A 1.2% agarose solution was heated and mixed 1:3 with medium containing 50% HBSS with Ca^2+^ and Mg^2+^ and 50% RPMI 1640 (20% heat-inactivated FBS). Three milliliters of the above-described solution was poured into a 35-mm culture dish and cooled gently. Punches 3.5 mm in diameter and 2.8 mm apart were made in wells according to the indicated experiments. The wells were then filled with 10 μL of neutrophil suspensions or chemoattractants. The gels were incubated for 2 h in a 37°C/5% CO_2_ incubator. Chemotaxis distances were observed and calculated at 100× magnification using a microscope.

### Flow cytometry

Neutrophils were stimulated with LPS 0.01 μg/mL in the presence or absence of inhibitors for 45 min and immediately washed twice with ice-cold PBS. Then, neutrophils were resuspended with PBS containing 5% FBS. Antibodies to FPR1 (BD, Cat. No. 556016, 1:5), C5aR (BD, Cat. No. 550494, 1:5), CXCR1 (BD, Cat. No. 551080, 1:5), CXCR2 (BD, Cat. No. 555933, 1:5) and BLTR1 (BD, Cat. No. 552836, 1:5) were added according to the manufacturer's instructions. After incubating for 45 min at 4°C, the membrane expression levels of chemoattractant receptors were detected by FCM.

### Laser confocal scanning microscope

Neutrophils were stimulated with 0.01 μg/mL LPS in the presence or absence of inhibitors for 45 min and immediately washed twice with ice-cold PBS. Two percent paraformaldehyde and 0.01% Triton X-100 were used for fixing and permeabilizing. The cells were blocked with 5% goat serum for 1 h. The primary antibody (1:200 dilution) and the secondary antibody (1:2000 dilution) were incubated sequentially. Neutrophil nuclei were stained with DAPI.

### Phospho-MAPK proteome array

Phospho-MAPK proteome array (ARY002B, R&D Systems, Minneapolis, MN, USA) was performed according to the manufacturer's instructions. Briefly, neutrophils were treated with or without 0.01 μg/mL LPS for 45 min. The neutrophils were then washed twice with ice-cold PBS and total protein was extracted using lysis buffer. 300-μg cell lysates were diluted, mixed with a cocktail of biotinylated detection antibodies, and incubated overnight with the Phospho-MAPK proteome array membrane at 4°C. Then, the membrane was washed with 1X Wash Buffer for 3 times and incubated with Streptavidin-HRP for 30 min at room temperature. The membrane was washed for 3 times, 1 mL of the Chemi Reagent Mix was pipetted evenly onto the membrane. Finally, the membrane was exposed to X-ray film for 3 minutes and the pixel densities in each spot of the array were determined by ImageJ software.

### Western blotting

Western blot was performed as described previously [33]. RIPA buffer that contained protease and phosphatase inhibitor cocktails was added for lysis of cells. The lysates were incubated with 3 × SDS buffer, boiled and loaded on 10% SDS–PAGE gels. 20 μg of protein were subjected to electrophoresis on 10% SDS polyacrylamide gels, with the use of the discontinuous system and transferred onto nitrocellulose membranes. The membranes were incubated with primary antibody and followed by secondary antibody conjugated to horseradish peroxidase (3:5000). ECL reagent was used to visualize bands with FluorChem FC3 (ProteinSimple, USA) and AlphaView 3.4.0 software was used for quantified analysis. Phosphorylated p38 (P-p38) was normalized to Total p38 (T-p38). Percent of control was presented.

### Statistics

All data were presented as the mean ± standard deviation. One-way factorial analysis of variance (ANOVA) and Tukey test for the comparisons were performed. A value of *P* < 0.05 was considered to be statistically significant.
